# A comparative study of the work-family conflicts prevalence, their sociodemographic, family, and work attributes, and their relation to the self-reported health status in Japanese and Egyptian civil workers

**DOI:** 10.1186/s12889-022-13924-0

**Published:** 2022-08-05

**Authors:** Omnyh Kamal Abd El Latief, Ehab Salah Eshak, Eman Mohamed Mahfouz, Hiroyasu Iso, Hiroshi Yatsuya, Eman Mohamed Sameh, Eman Ramadan Ghazawy, Sachiko Baba, Shimaa Anwer Emam, Ayman Soliman El-khateeb, Ebtesam Esmail Hassan

**Affiliations:** 1 Public Health Department, Faculty of Medicine, Minia University, El-Minia, Egypt; 2 Public Health, Department of Social Medicine, Graduate School of Medicine, Osaka University, Osaka, Japan; 3 Department of Public Health and Health System, Graduate School of Medicine, Nagoya University, Nagoya, Japan; 4 Bioethics and Public Policy, Department of Social Medicine, Osaka University Graduate School of Medicine, Osaka, Japan

**Keywords:** Cross-cultural study, Work-family conflict, Self-rated health, Gender, Civil workers, Japan, Egypt

## Abstract

**Background:**

Cross-cultural studies studying work-family conflicts (W_F_Cs) are scarce. We compared the prevalence of W_F_Cs, factors correlated with them, and their association with self-rated health between Japan and Egypt.

**Methods:**

Among 4862 Japanese and 3111 Egyptian civil workers recruited by a convenience sample in 2018/2019 and reported self-rated health status, we assessed the W_F_Cs by the Midlife Development in the US (MIDUS) and attributed them to sociodemographic, family, and work variables. We also evaluated the W_F_Cs’ gender- and country-specific associations with self-rated health by logistic regression analyses.

**Results:**

W_F_Cs were more prevalent in Egyptian than in Japanese women (23.7% vs. 18.2%) and men (19.1% vs. 10.5%), while poor self-rated health was more prevalent in Japanese than Egyptians (19.3% and 17.3% vs. 16.9% and 5.5%). Longer working hours, shift work, and overtime work were positively associated with stronger work-to-family conflict (WFC). Whereas being single was inversely associated with stronger family-to-work conflict (FWC). Living with children, fathers, or alone in Japan while education in Egypt was associated with these conflicts. The OR (95% CI) for poor self-reported health among those with the strong, in reference to weak total W_F_Cs, was 4.28 (2.91–6.30) and 6.01 (4.50–8.01) in Japanese women and men and was 2.46 (1.75–3.47) and 3.11 (1.67–5.80) in Egyptian women and men.

**Conclusions:**

Japanese and Egyptian civil workers have different prevalence and correlated factors of W_F_Cs and self-rated health. W_F_Cs were associated in a dose–response pattern with poor-self-rated health of civil workers in both countries.

**Supplementary Information:**

The online version contains supplementary material available at 10.1186/s12889-022-13924-0.

## Introduction

Work and family are key realms of human life. However, sometimes the individuals’ time, strain, and behavior related to one realm clash with those of the other [1]. Unfair distribution of the subject’s energy and time could lead to some sort of conflict [2]. This conflict could be directed from work to family and described as work-to-family conflict (WFC) or from family to work and described as family-to-work conflict (FWC) [3]. Both WFC and FWC compose the total work-family conflicts (W_F_Cs*) *[4, 5]*. Khan *et al*. (1964)* have defined W_F_Cs as “inter-role conflicts in which the role pressures from the work and family domains are mutually incompatible in some respect” [6].

W_F_Cs have become a rich area for organizational, social, and health research because they influence organizational achievement [7] and workers' personal lives [8, 9]. W_F_Cs have been related to absenteeism, tardiness, leaving work early, turnover intentions, and other negative work behaviors [10, 11]. Meanwhile, W_F_Cs have been associated with adverse physical and mental health outcomes [9, 11–15].

The World Health Organization (WHO) suggested in the early 1990s the implementation of self-rated health as a valuable tool for assessing individuals' health and quality of life [16]. Since then, self-rated health has been widely used in social science research.

So far, there is a considerable bulk of research studied the attributes of W_F_Cs [17–21] and linked W_F_Cs to poor self-rated health of community dwellers [8, 12] and working populations [13, 22–24]. The literature indicated vast variabilities in the W_F_Cs’ levels and their correlates, the proportions of subjects with poor self-rated health, and the magnitude of association between these conflicts and self-rated health across different cultures and populations. Yet, cross-cultural studies that compare the attributes and the health sequences of W_F_Cs among working people of different cultures are limited [5]. In Egypt and Japan, the published literature was based on small sample studies and indicated social and occupational variabilities between the two populations, such as the differences in the family structure and the average daily working hours. Yet, the two countries are alike in terms of the lifetime-employment system and the community’s view of males as breadwinners and females as caregivers. The Egyptian studies suggested the prevalence of WFC, FWC, and poor self-rated health at 46.7%, 50.8%, and 16.9% [12]. The prevalence reported in the Japanese studies ranged between 15.2% to 54.0% for WFC, 21.2 to 36.4% for FWC, and 13.9% to 35.2% for poor self-rated health in men and 22.8% to 72.5%, 16.3% to 56.8%, and 17.7% to 36.0%, respectively in women [8, 13, 20]. Accordingly, in the current research, we aimed to run a cross-cultural study among large samples of Egyptian and Japanese civil workers to compare the prevalence and correlated factors of the W_F_Cs and poor self-rated health in the two working populations and to compare the associations of W_F_Cs with the poor self-rated health among the civil workers in both countries.

## Methods

### Subjects

This comparative, cross-cultural study data were collected separately for Japanese and Egyptian civil workers who work in a central prefecture/governorate (Aichi in Japan and Minia in Egypt). A total of 5310 civil workers aged 20–60 years responded to the 2018 data collection cycle of the Aichi Workers' Cohort study, and 3133 Egyptian civil workers of the same age range responded to the Minia University Public Health Department’s survey in 2019. The Aichi Workers' Cohort study [25] and the Egyptian survey were published previously [26]. As we aim to study the work and family interface, we excluded civil workers who were not living with a spouse, children, parents, or other relatives on the condition they reported the number of family members = 0; thus, the final sample consisted of 4862 Japanese and 3111 Egyptian civil workers. The ethical review boards at Nagoya University, Japan, and Minia University, Egypt, have approved each survey. The ethical review board in [Masked for Review] (which hosted the Japanese and Egyptian collected data) has also approved the comparative study (approval no 19501). All Egyptian participants consented to provide their data for the comparative research, and Japanese participants who did not respond to an opt-out consent were considered agreeing to be involved in the comparative study.

### Data

The paper–pencil self-administered questionnaire used in both countries contained the same set of targeted variables, including information on the civil workers' sociodemographic, family, job, and health aspects.

#### Work-family conflicts

The following four statements were used to investigate the level of FWC, 1- “Thinking about home troubles can confuse you at work,” 2- “The work time is reduced due to home-related issues,” 3- “Your own time to relax is reduced due to responsibilities at home,” and 4- “ Due to housework, you cannot have enough sleeping time you need to accomplish your work.” The following four statements were used to investigate the level of work-to-family conflict (WFC), 1- “Work problems make you annoyed at home,” 2- “I dedicate less time to my family because I have to work,” 3- “My work depletes my energy that I feel not able to pay attention to anything at home,” and 4- “I am often out of home for a long time due to work needs.” For each statement, participants can choose one frequency response on a three-point Likert scale (0 = never, 1 = to some extent, 2 = often/ very often) as initially indicated by the Midlife Development in the United States National Study [27] and used in previous Japanese [8] and Egyptian [28] settings.

#### Health status (Self-reported)

The participants were asked to choose either “1 = very good, 2 = fairy good, 3 = good, 4 = not very good, 5 = not good” in response to the question “How do you rate your current health status?”. Participants who chose “not very good” and “not good” were considered to have a poor self-reported health status.

#### Other variables

We collected information on the sociodemographic, family, and work attributes of the participants, which we believe it could relate to the W_F_Cs, such as age, gender, marital status, education, occupation, living arrangement, number of family members, and how children were under the age of 14 years, the number of average working hours per day, working overtime or additional job, time for one-way commuting to work, and whether the work is a regular day time work or requires night shifts. We also ascertained the participants’ lifestyles by inquiring about their smoking and drinking habits. We converted physical activity into the metabolic equivalent of task (METs) unit according to the self-reported hours spent in different activities.

### Statistical analysis

We showed the descriptive analyses of the collected data, gender-specific to each country, as mean (SD) or proportion, and included the frequency responses to each statement of the FWC and WFC. The FWC and WFC scores ranged between 0 and 8 points, and both were combined to create the total W_F_Cs score, which ranged between 0 and 16 points, as indicated by previous studies [4, 5, 28].

We used the logistic regression analyses to assess the gender- and country-specific associations of sociodemographic, family, and work factors with the different levels of FWC and WFC [weak conflict level (< 2 points), moderate conflict level (2–4 points), and strong conflict level (≥ 5 points)]. Participants should have been ranked strong in at least one form of conflict (FWC and/or WFC) while ranked moderate in the other form of conflict to be in the strong total W_F_Cs category. On the other hand, participants should have been ranked weak in at least one form of conflict while ranked moderate in the other form of conflict to be in the weak total W_F_Cs category. Other than these, participants were ranked moderate in the total W_F_Cs [14, 29]. We computed the odds ratios (ORs) and 95% confidence intervals (CIs) of having poor self-reported health across the increasing categories of the conflicts, age groups, smoking status, alcohol drinking status (for Japanese only), and quartiles of physical activity using the weak category of conflict, age ≤ 30 years, never smokers, never drinkers, and the lowest quartile of physical activity respectively as the reference groups. The logistic regression models were adjusted for socioeconomic-, family-, and work-related factors.

We used the SAS software version 9.4 (SAS Institute Inc, Cary, NC, USA) for the analyses considering a two-tailed *p*-value < 0.05 for statistical significance.

## Results

### Descriptive analyses in Tables 1 and 2

**Table 1:** Descriptive analyses of the study variables among Japanese and Egyptian civil workers

	**Japan**		**Egypt**	
	Women	Men	Women	Men
Age, years^1^	38.8 (11.0)	44.2 (10.8)	38.4 (10.1)	43.3 (10.3)
Education				
Junior high/High	80 (5.0)	201 (6.1)	218 (13.5)	383 (25.6)
Vocational	501 (31.4)	217 (6.7)	305 (18.9)	354 (23.6)
University or more	1013 (63.6)	2847 (87.2)	1091 (67.6)	760 (50.8)
Marital status				
Married	925 (58.1)	2454 (75.2)	1258 (77.9)	1353 (90.4)
Divorced/Separated	77 (4.8)	68 (2.1)	42 (2.6)	12 (0.8)
Widow	13 (0.8)	17 (0.5)	79 (4.9)	10 (0.7)
Single	578 (36.3)	725 (22.2)	235 (14.6)	122 (8.2)
Cohabitants				
Spouse	905 (56.7)	2428 (74.3)	1210 (75.0)	1283 (85.7)
Children	707 (44.3)	1895 (58.0)	1123 (69.6)	1212 (81.0)
Father	394 (24.7)	622 (19.0)	232 (14.4)	250 (16.7)
Mother	500 (31.3)	851 (26.1)	292 (18.1)	334 (22.3)
Others	275 (17.2)	295 (9.0)	126 (7.8)	87 (5.8)
Alone	176 (11.0)	248 (7.6)	10 (0.6)	13 (0.8)
Number of family members^1^	2.2 (1.4)	2.4 (1.4)	3.1 (1.9)	3.9 (1.8)
Number of children < 14 years old^1^	0.7 (0.9)	0.7 (0.9)	1.1 (1.2)	1.4 (1.4)
Occupation				
Professional	861 (54.0)	1424 (43.6)	1070 (66.3)	715 (47.8)
Clerks	735 (46.0)	1842 (56.4)	374 (23.2)	521 (34.8)
Technical/worker	––––	––––	170 (10.5)	260 (17.4)
Regular daytime work				
Yes	1201 (75.4)	2977 (91.2)	1352 (83.8)	1165 (77.8)
No	392 (24.6)	286 (8.8)	262 (16.2)	332 (22.2)
Job hours per day^1^	8.2 (1.3)	8.4 (1.1)	6.8 (1.5)	7.5 (1.7)
Time to reach work, minutes^1^	45.8 (24.0)	56.4 (26.2)	30.0 (21.7)	31.1 (21.0)
Working overtime/Extra job				
No	1394 (87.3)	2797 (85.6)	1396 (86.5)	1396 (86.5)
Yes	202 (12.7)	469 (14.4)	218 (13.5)	218 (13.5)
Family-to-work conflict				
Weak (score < 2)	746 (46.7)	1874 (57.4)	690 (42.8)	773 (51.6)
Moderate (score 2–4)	611 (38.3)	1137 (34.8)	717 (44.4)	577 (38.6)
Strong (> = 5)	239 (15.0)	255 (7.5)	207 (12.8)	147 (9.8)
Family-to-work conflict score^1^	2.2 (2.1)	1.6 (1.9)	3.1 (2.1)	2.6 (2.1)
Work-to-family conflict (WFC)				
Weak (Score < 2)	562 (35.2)	1428 (43.7)	557 (34.5)	563 (37.6)
Moderate (score 2–4)	835 (52.3)	1593 (48.8)	664 (41.1)	592 (39.6)
Strong (> = 5)	199 (12.5)	245 (7.5)	393 (24.4)	342 (22.8)
Work-to-family conflict score^1^	2.3 (1.8)	2.0 (1.7)	3.7 (2.4)	3.5 (2.4)
Total work-family conflicts (W_F_Cs)				
Weak	859 (53.8)	2103 (64.4)	752 (46.6)	787 (52.6)
Moderate	447 (28.0)	821 (25.1)	479 (29.7)	424 (28.3)
Strong	290 (18.2)	342 (10.5)	383 (23.7)	286 (19.1)
Total work-family conflict score^1^	4.5 (3.3)	3.6 (3.0)	6.8 (3.7)	6.1 (3.8)
Self-reported health				
Good	1288 (80.7)	2703 (82.8)	1341 (83.1)	1414 (94.5)
Poor	308 (19.3)	563 (17.3)	273 (16.9)	83 (5.5)

^1^Mean (SD), all such variables. Other variables in the table were presented as numbers (proportions)

**Table 2:** Comparing the response frequency to the work-family conflict items in Japanese and Egyptian civil workers

	**Japan**			**Egypt**		
	Never *n* (%)	Sometimes *n* (%)	Often *n* (%)	Never *n* (%)	Sometimes *n *(%)	Often/always *n* (%)
**Women**						
**Family-to-work conflict (FWC)**						
“Thinking about home troubles can confuse you at work”	1006 (63.0)	488 (30.6)	102 (6.4)	1045 (64.7)	443 (27.5)	126 (7.8)
“The work time is reduced due to home-related issues”	944 (59.1)	595 (37.3)	57 (3.6)	711 (44.0)	684 (42.4)	219 (13.6)
“Your own time to relax is reduced due to responsibilities at home”	847 (53.0)	566 (35.5)	183 (11.5)	657 (40.7)	610 (37.8)	347 (21.5)
“Due to housework, you cannot have enough sleeping time you need to accomplish your work”	756 (47.4)	542 (33.9)	298 (18.7)	417 (25.8)	577 (35.8)	620 (38.4)
**Work-to-Family conflict (WFC)**						
“Work problems make you annoyed at home”	524 (32.8)	734 (46.0)	338 (21.2)	505 (32.3)	658 (40.8)	451 (27.9)
“I dedicate less time to my family because I have to work”	654 (41.0)	755 (47.3)	187 (11.7)	451 (27.9)	663 (41.1)	500 (40.0)
“My work depletes my energy that I feel not able to pay attention to anything at home”	1303 (81.6)	279 (17.5)	14 (0.9)	793 (49.1)	457 (28.3)	364 (22.6)
“I am often out of home for long time due to work need”	844 (52.9)	600 (37.6)	152 (9.5)	531 (32.9)	612 (37.9)	471 (29.2)
**Men**						
**Family-to-work conflict (FWC)**						
“Thinking about home troubles can confuse you at work”	2213 (67.7)	949 (29.1)	104 (3.2)	938 (62.7)	415 (27.7)	144 (9.6)
“The work time is reduced due to home-related issues”	2059 (63.1)	1118 (34.2)	89 (2.7)	727 (48.6)	547 (36.5)	223 (14.9)
“Your own time to relax is reduced due to responsibilities at home”	2383 (73.0)	749 (22.9)	134 (4.1)	766 (51.2)	512 (34.2)	219 (14.6)
“Due to housework, you cannot have enough sleeping time you need to accomplish your work”	1733 (53.0)	1194 (36.6)	339 (10.4)	584 (39.0)	577 (38.5)	336 (22.5)
**Work-to-Family conflict (WFC)**						
“Work problems make you annoyed at home”	1256 (38.5)	1483 (45.4)	527 (16.1)	525 (35.1)	518 (34.6)	454 (30.3)
“I dedicate less time to my family because I have to work”	1694 (51.9)	1368 (41.9)	204 (6.2)	556 (37.1)	572 (38.2)	369 (24.7)
“My work depletes my energy that I feel not able to pay attention to anything at home”	2632 (80.6)	598 (18.3)	36 (1.1)	637 (42.6)	454 (30.3)	406 (27.1)
“I am often out of home for long time due to work need”	1976 (60.5)	1103 (33.8)	187 (5.7)	595 (39.8)	532 (35.5)	370 (24.7)

In both countries, most of the participants were educated at a university level or above, currently married, and working regular daytime jobs. There were more singles and living alone Japanese than Egyptian civil workers. Larger families were seen in Egypt than in Japan. The Japanese civil workers worked on average 8.2 h for females and 8.4 h for males per day, and 12.7% and 14.4% of them worked overtime. On the other hand, the average working hours per day and the proportion of civil workers who worked additional jobs in Egypt were 6.8 h and 13.5% for females and 7.5 h and 44.8% for males.

Japanese women had a higher prevalence of FWC (15.0%) than Egyptian women (12.8%), but the opposite was found in men (7.8% in Japanese men and 9.8% in Egyptian men). A higher prevalence of strong total W_F_Cs was seen in Egypt (23.7% in women and 19.1% in men) than in Japan (18.2% in women and 10.5% in men). There were always higher proportions of Egyptians selecting the highest frequency option “often” in responding to each conflict statement than the Japanese counterparts.

The prevalence of poor self-rated health among the Japanese civil workers was 19.3% in females and 17.3% in males. In contrast, the Egyptian civil workers reported a lower prevalence, 16.9% in females and 5.5% in males.

The gender-and country-specific participants’ characteristics according to the categories of total W_F_Cs levels are shown in Supplemental Table 1. There were significant differences in the family-related factors across increasing categories of total W_F_Cs in the Japanese but not the Egyptian women and men. On the other hand, the work-related factors showed similar variations in both genders of both countries.

### Logistic regression for factors predicting the conflicts’ score in Table 3

**Table 3:** Multivariable binominal logistic regression for factors associated with conflicts between work and family in Japanese and Egyptian women and men

	**Japanese**				**Egyptian**			
	**Women**		**Men**		**Women**		**Men**	
	**FWC**	**WFC**	**FWC**	**WFC**	**FWC**	**WFC**	**FWC**	**WFC**
	**OR (95% CI)**	**OR (95% CI)**	**OR (95% CI)**	**OR (95% CI)**	**OR (95% CI)**	**OR (95% CI)**	**OR (95% CI)**	**OR (95% CI)**
**Age**	1.01 (0.99–1.03)	1.01 (0.99–1.03)	0.96 (0.90–0.99)	0.99 (0.98–1.01)	0.96 (0.95–0.98)	0.95 (0.93–0.97)	0.97 (0.96–0.99)	0.96 (0.94–0.98)
**Education**								
> = university	1.00	1.00	1.00	1.00	1.00	1.00	1.00	1.00
Junior high/ High	0.74 (0.39–1.40)	1.56 (0.83–2.95)	0.82 (0.58–1.15)	0.84 (0.60–1.17)	0.53 (0.37–0.77)	0.69 (0.47–0.98)	0.82 (0.59–1.14)	0.65 (0.46–0.91)
Vocational	0.75 (0.53–1.08)	0.98 (0.71–1.43)	0.93 (0.67–1.31)	0.88 (0.63–1.23)	0.85 (0.62–1.16)	0.98 (0.73–1.41)	0.98 (0.74–1.35)	0.71 (0.52–0.96)
**Marital status**								
Married	1.00	1.00	1.00	1.00	1.00	1.00	1.00	1.00
Divorced	1.07 (0.20–3.02)	0.91 (0.40–4.24)	0.23 (0.02–2.37)	0.81 (0.19–0.97)	0.57 (0.23–1.41)	0.80 (0.32–2.00)	1.37 (0.34–5.54)	0.62 (0.18–2.17)
Widow	0.30 (0.06–1.55)	0.43 (0.09–2.02)	1.06 (0.45–6.60)	0.89 (0.13–1.56)	0.55 (0.25–1.21)	0.57 (0.25–1.27)	0.45 (0.11–1.18)	0.26 (0.05–1.36)
Single	0.31 (0.09–0.96)	0.93 (0.30–2.83)	0.20 (0.02–0.87)	0.74 (0.62–0.87)	0.34 (0.15–0.77)	0.51 (0.22–1.17)	0.56 (0.27–0.98)	1.30 (0.86–3.02)
**Living with spouse**	0.52 (0.15–1.76)	0.90 (0.32–2.52)	0.35 (0.04–3.19)	0.81 (0.60–1.95)	0.69 (0.37–1.31)	0.73 (0.39–1.38)	1.13 (0.69–1.63)	0.94 (0.72–1.98)
**Living with children**	2.10 (1.25–3.54)	0.94 (0.61–1.77)	1.63 (1.19–2.23)	0.83 (0.60–1.14)	1.14 (0.84–1.55)	0.94 (0.68–1.28)	0.92 (0.76–1.63)	1.54 (0.98–2.26)
**Living with father**	0.28 (0.15–0.51)	0.31 (0.16–0.57)	0.79 (0.55–1.11)	0.98 (0.69–1.39)	0.72 (0.44–1.17)	0.92 (0.54–1.55)	1.11 (0.74–1.67)	0.73 (0.48–1.10)
**Living with mother**	1.65 (0.86–3.15)	1.45 (0.76–2.79)	1.12 (0.81–1.54)	0.85 (0.62–1.16)	1.15 (0.70–1.89)	1.25 (0.75–2.09)	1.30 (0.90–1.88)	1.24 (0.85–1.81)
**Living with others**	0.76 (0.43–1.14)	0.40 (0.23–1.69)	0.83 (0.55–1.81)	0.62 (0.42–1.12)	1.36 (1.03–2.44)	1.76 (0.83–2.74)	0.96 (0.58–1.60)	1.32 (0.75–2.30)
**Living alone**	0.64 (0.36–0.82)	0.88 (0.67–1.16)	0.61 (0.39–0.84)	0.82 (0.74–0.93)	1.78 (0.48–6.67)	1.20 (0.30–4.85)	1.70 (0.42–6.89)	1.70 (0.38–7.51)
**Number of family members**	0.94 (0.82–1.34)	1.48 (1.18–1.86)	0.97 (0.85–1.11)	1.10 (0.97–1.25)	1.07 (1.00–1.14)	1.04 (0.97–1.12)	1.02 (0.89–1.15)	1.01 (0.93–1.10)
**Children < 14 years old**	1.78 (1.36–2.33)	1.14 (0.88–1.47)	1.54 (1.35–1.76)	1.22 (1.07–1.38)	1.03 (0.91–1.12)	1.01 (0.91–1.13)	1.03 (0.93–1.14)	1.03 (0.94–1.15)
**Occupation**								
Professional	1.00	1.00	1.00	1.00	1.00	1.00	1.00	1.00
Clerks	0.97 (0.84–1.63)	0.59 (0.43–0.80)	1.22 (0.93–1.45)	0.99 (0.83–1.17)	0.94 (0.71–1.26)	0.76 (0.56–0.98)	1.15 (0.89–1.50)	1.05 (0.81–1.38)
Technical/ manual	––	––	––	––-	0.80 (0.53–1.20)	0.65 (0.42–1.03)	1.40 (0.95–2.07)	1.40 (0.95–2.07)
**Not regular daytime work**	1.18 (0.78–1.81)	2.05 (1.31–3.22)	1.70 (1.21–2.40)	1.75 (1.25–2.47)	1.19 (0.86–1.66)	3.25 (2.23–4.72)	1.22 (0.89–1.68)	1.41 (1.02–1.96)
**Job hours per day**	1.07 (0.86–1.16)	1.37 (1.22–1.54)	1.07 (1.00–1.16)	1.22 (1.13–1.32)	1.07 (0.97–1.15)	1.19 (1.09–1.29)	1.18 (1.09–1.28)	1.16 (1.08–1.26)
**Time to reach work**	0.92 (0.76–1.12)	1.08 (0.62–1.36)	0.90 (0.80–1.22)	1.10 (1.02–1.19)	1.18 (0.91–1.57)	1.18 (1.03–1.31)	0.94 (0.79–1.39)	1.17 (1.02–1.28)
**Working overtime/extra job**	1.10 (0.99–1.75)	5.56 (2.89–8.70)	1.51 (1.17–1.96)	4.17 (3.08–5.65)	1.71 (1.24–2.36)	1.93 (1.39–2.70)	1.70 (1.34–2.15)	2.19 (1.73–2.78)

The multivariable binominal logistic regression model simultaneously included all the variables in the table

#### FWC

In Japanese women and men, living with children was positively associated, while living with a father or alone was inversely related to the FWC. The number of children in the family was related to the FWC in Japanese women; OR = 2.10: 95% CI = 1.25–3.54. Meanwhile, overtime work in both women (OR = 1.10: 95% CI = 0.99–1.75) and men (OR = 1.51: 95% CI = 1.17–1.96), and job hours per day (OR = 1.07: 95% CI = 1.00–1.16) and shiftwork (OR = 1.70: 95% CI = 1.21–2.40) in Japanese men were positively associated with the FWC.

On the other hand, working an extra job in Egyptian women (OR = 1.71: 95% CI = 1.24–2.36) and men (OR = 1.70: 95% CI = 1.34–2.15) was associated with the FWC. Junior high/high school education, in reference to university education or more (OR = 0.53: 95% CI = 0.37–0.77) and living with other family members (OR = 1.36: 95% CI = 1.03–2.44) in women were associated with the FWC; while job hours in men was related to the FWC (OR = 1.18: 95% CI = 1.09–1.28).

#### WFC

In reference to professionals, the Japanese female clerks had an OR (95% CI) of WFC = 0.59 (0.43–0.80). Shift work, job hours per day, overtime work in Japanese women and men, and commuting time to work in Japanese men were associated with the WFC. Japanese women living with fathers had lower odds of WFC (OR = 0.31: 95% CI = 0.16–0.57). Single, divorced, and living alone Japanese men had lower odds of WFC, while the OR (95%CI) of Japanese men’s WFC with the increasing number of family members was 1.22 (1.07–1.38).

The level of WFC in Egyptian women and men was positively associated with shift work, job hours per day, working an extra job, and commuting time to work, while inversely associated with the education level. The OR (95%CI) of the WFC in Egyptian female clerks in reference to professionals was 0.76 (0.56–0.98).

### Logistic regression for the association between the conflicts and self-reported health in Table 4

**Table 4:** Odds ratios and (95%CIs) for poor self-rated health in Japanese and Egyptian civil workers according to gender-and country-specific categories of conflicts between work and family

	**Japan**				**Egypt**			
	Case/total	Model 1	Model 2	Model 3	Case/total	Model 1	Model 2	Model 3
**Women**	308/1596				273/1614			
Family-to-work conflict (***FWC)***								
Weak	116/746	1.00	1.00	1.00	95/690	1.00	1.00	1.00
Moderate	115/611	1.34 (0.99–2.81)	1.59 (1.15–2.20)	1.61 (1.16–2.24)	121/717	1.33 (0.99–1.79)	1.32 (0.97–1.77)	1.30 (0.96–1.76)
Strong	77/239	2.91 (2.02–4.21)	4.05 (2.65–6.18)	4.08 (2.66–6.27)	57/207	2.59 (1.77–3.79)	2.62 (1.77–3.86)	2.57 (1.73–3.82)
*p*-trend		< 0.0001	< 0.0001	< 0.0001		< 0.0001	< 0.0001	< 0.0001
*Work-to-family conflict (* ***WFC)***								
Weak	67/562	1.00	1.00	1.00	70/557	1.00	1.00	1.00
Moderate	171/835	2.04 (1.49–2.80)	2.04 (1.48–2.81)	2.20 (1.58–3.07)	114/664	1.60 (1.15–2.23)	1.63 (1.17–2.27)	1.61 (1.14–2.26)
Strong	70/199	4.56 (3.05–6.84)	4.95 (3.27–7.49)	5.45 (3.47–8.54)	89/393	2.46 (1.71–3.54)	2.59 (1.79–3.75)	2.63 (1.79–3.88)
*P*-trend		< 0.0001	< 0.0001	< 0.0001		< 0.0001	< 0.0001	< 0.0001
***Total work-family conflicts (W–F–Cs)***								
Weak	122/859	1.00	1.00	1.00	101/752	1.00	1.00	1.00
Moderate	92/447	1.73 (1.26–2.37)	1.88 (1.35–2.61)	1.88 (1.34–2.62)	77/479	1.34 (0.96–1.85)	1.36 (0.98–1.90)	1.37 (0.98–1.92)
Strong	94/290	3.36 (2.39–4.73)	4.19 (2.89–6.08)	4.28 (2.91–6.30)	95/383	2.43 (1.75–3.36)	2.47 (1.78–3.44)	2.46 (1.75–3.47)
*p*-trend		< 0.0001	< 0.0001	< 0.0001		< 0.0001	< 0.0001	< 0.0001
**Age**								
< = 30 years	99/505	1.00	1.00	1.00	69/454	1.00	1.00	1.00
31–40 years	58/316	0.64 (0.43–0.94)	0.79 (0.51–1.20)	0.79 (0.49–1.16)	80/530	1.10 (0.77–1.58)	1.09 (0.72–1.63)	1.08 (0.71–1.65)
41–50 years	94/502	0.65 (0.46–0.92)	0.84 (0.57–1.26)	0.77 (0.51–1.16)	70/379	1.40 (0.96–2.05)	1.31 (0.84–2.05)	1.32 (0.83–2.09)
> 50 years	57/273	0.89 (0.59–1.33)	0.98 (0.60–1.61)	0.91 (0.55–1.51)	54/251	1.83 (1.20–2.77)	1.48 (0.89–2.46)	1.51 (0.89–2.56)
*p*-trend		0.41	0.92	0.92		0.002	0.09	0.08
**Smoking habit**								
Never	288/1504	1.00	1.00	1.00	272/1604	1.00	1.00	1.00
Former	14/59	1.25 (0.66–2.38)	1.23 (0.63–2.39)	1.26 (0.63–2.52)	1/5	1.40 (0.15–12.8)	1.39 (0.15–13.0)	1.26 (0.13–12.2)
Current	6/33	0.99 (0.40–2.48)	0.92 (0.37–2.31)	1.02 (0.40–2.59)	0/5	––––-	––––-	––––-
*p*-trend		0.67	0.60	0.60		0.49	0.49	0.47
**Alcohol drinking habit**								
Never	200/1065	1.00	1.00	1.00	NA	NA	NA	NA
Former	10/32	0.93 (0.71–1.23)	0.90 (0.68–1.20)	0.90 (0.68–1.21)	NA	NA	NA	NA
Current	98/499	1.69 (0.75–3.81)	2.02 (0.88–4.67)	2.06 (0.88–4.78)	NA	NA	NA	NA
*p*-trend		0.62	0.40	0.40		NA	NA	NA
**Physical activity in MET units**								
Q1	44/258	1.00	1.00	1.00	60/361	1.00	1.00	1.00
Q2	53/325	1.02 (0.64–1.61)	1.00 (0.71–1.70)	1.01 (0.72–1.71)	58/340	1.12 (0.76–1.65)	1.10 (0.74–1.63)	1.06 (0.71–1.58)
Q3	44/286	0.92 (0.79–1.22)	0.90 (0.77–1.21)	0.90 (0.76–1.23)	43/237	0.99 (0.69–1.44)	0.99 (0.68–1.44)	0.95 (0.65–1.40)
Q4	41/228	1.10 (0.82–1.54)	1.08 (0.78–1.45)	1.09 (0.78–1.44)	52/285	0.81 (0.56–1.16)	0.79 (0.55–1.14)	0.77 (0.53–1.12)
*p*-trend		0.45	0.67	0.65		0.46	0.38	0.32
**Men**	563/3266				83/1497			
Family-to-work conflict (***FWC)***								
Weak	232/1874	1.00	1.00	1.00	15/563	1.00	1.00	1.00
Moderate	242/1137	1.99 (1.63–2.44)	2.47 (1.99–3.07)	2.38 (1.91–2.96)	32/592	1.19 (0.72–1.96)	1.18 (0.71–1.98)	1.15 (0.68–1.94)
Strong	89/255	3.95 (2.93–5.34)	5.35 (3.88–7.47)	5.16 (3.73–7.15)	36/342	2.87 (1.55–5.33)	3.09 (1.64–5.78)	2.77 (1.45–5.30)
*p*-trend		< 0.0001	< 0.0001	< 0.0001		< 0.0001	< 0.0001	< 0.0001
*Work-to-family conflict (* ***WFC)***								
Weak	143/1428	1.00	1.00	1.00	36/773	1.00	1.00	1.00
Moderate	310/1593	2.22 (1.79–2.75)	2.48 (1.99–3.09)	2.35 (1.88–2.95)	30/577	2.21 (1.18–4.16)	2.32 (1.22–4.42)	2.33 (1.21–4.47)
Strong	110/245	7.51 (5.51–10.24)	9.05 (6.56–12.49)	8.45 (6.01–11.87)	17/147	5.01 (2.63–9.54)	5.37 (2.78–9.40)	5.06 (2.52–10.17)
*P*-trend		< 0.0001	< 0.0001	< 0.0001		< 0.0001	< 0.0001	0.009
***Total work-family conflicts (W–F–Cs)***								
Weak	252/2103	1.00	1.00	1.00	25/787	1.00	1.00	1.00
Moderate	179/821	2.12 (1.71–2.63)	2.51 (2.00–3.14)	2.35 (1.87–2.95)	32/424	2.80 (1.62–4.84)	2.84 (1.63–4.95)	2.79 (1.58–4.92)
Strong	132/342	4.85 (3.73–6.29)	6.36 (4.81–8.41)	6.01 (4.50–8.01)	26/286	3.26 (1.82–5.84)	3.47 (1.92–6.29)	3.11 (1.67–5.80)
*P*-trend		< 0.0001	< 0.0001	< 0.0001		< 0.0001	< 0.0001	< 0.0001
**Age**								
< = 30 years	94/559	1.00	1.00	1.00	12/202	1.00	1.00	1.00
31–40 years	90/512	0.72 (0.52–1.01)	1.08 (0.75–1.55)	1.07 (0.75–1.54)	18/448	0.66 (0.31–1.42)	1.09 (0.42–2.78)	1.03 (0.39–2.67)
41–50 years	193/1081	0.83 (0.62–1.10)	1.40 (1.00–1.94)	1.40 (1.00–1.96)	29/387	1.45 (0.70–2.98)	2.40 (0.91–6.37)	2.47 (0.92–6.64)
> 50 years	186/1114	0.88 (0.66–1.18)	1.39 (0.98–1.98)	1.48 (1.03–2.13)	24/460	1.01 (0.47–2.15)	1.51 (0.53–4.25)	1.47 (0.51–4.23)
*p*-trend		0.58	0.009	0.009		0.44	0.43	0.45
**Smoking habit**								
Never	364/2108	1.00	1.00	1.00	46/916	1.00	1.00	1.00
Former	126/752	1.00 (0.79–1.26)	1.09 (0.85–1.38)	1.09 (0.86–1.40)	23/443	1.08 (0.47–1.83)	1.02 (0.60–1.75)	1.02 (0.59–1.75)
Current	73/406	1.09 (0.81–1.45)	1.10 (0.82–1.48)	1.13 (0.84–1.52)	14/138	2.22 (1.15–4.29)	2.24 (1.15–4.36)	2.27 (1.16–4.45)
*p*-trend		0.63	0.43	0.56		0.07	0.09	0.08
**Alcohol drinking habit**								
Never	450/2712	1.00	1.00	1.00	NA	NA	NA	NA
Former	14/52	0.82 (0.64–1.80)	0.85 (0.66–1.10)	0.84 (0.65–1.09)	NA	NA	NA	NA
Current	99/502	1.50 (0.76–2.95)	1.55 (0.78–3.09)	1.60 (0.80–3.20)	NA	NA	NA	NA
*p*-trend		0.52	0.65	0.65		NA	NA	NA
**Physical activity in MET units**								
Q1	76/500	1.00	1.00	1.00	16/276	1.00	1.00	1.00
Q2	88/627	0.99 (0.76–1.28)	0.98 (0.75–1.28)	0.98 (0.75–1.28)	13/280	1.20 (0.61–2.35)	1.23 (0.63–2.42)	1.21 (0.61–2.40)
Q3	96/572	1.20 (0.90–1.58)	1.20 (0.90–1.59)	1.18 (0.89–1.58)	14/210	0.79 (0.43–1.48)	0.77 (0.41–1.45)	0.76 (0.40–1.45)
Q4	82/419	0.92 (0.70–1.20)	0.91 (0.70–1.20)	0.90 (0.61–1.39)	16/345	0.90 (0.50–1.63)	0.94 (0.51–1.63)	0.95 (0.51–1.75)
*p*-trend		0.17	0.20	0.20		0.67	0.73	0.71

Model 1: Adjusted for education

Model 2: Adjusted further for family factors (marital status, family structure, number of family members and the presence of family members below 14 years old)

Model 3: Adjusted further for work factors (occupation, job hours per day, time to commute to work, shift work, working overtime or extra job)

In a model that included W_F_Cs, age, smoking, alcohol drinking, and physical activity and adjusted for education as a socioeconomic factor, we found dose–response positive associations between increasing total W_F_Cs and its two forms with poor self-reported health status in Japanese and Egyptian civil workers (*p*-trend < 0.001). Adjusting for family-related factors in model 2 augmented the association between FWC and self-reported health among Japanese but not Egyptian women and men. The observed associations did not change materially after further controlling for the job-related factors in Model 3; the multivariable ORs (95% CIs) of poor health for the moderate and strong categories of FWC, in reference to the weak category, were 1.61 (1.16–2.24) and 4.08 (2.66–6.27) in Japanese women, 1.30 (0.96–1.76) and 2.57 (1.73–3.82) in Egyptian women, 2.38 (1.91–2.96) and 5.16 (3.73–7.15) in Japanese men, and 1.15 (0.68–1.94) and 2.77 (1.45–5.30) in Egyptian men. The respective ORs (95% CIs) for the moderate and strong categories of WFC, in reference to the weak category, were 2.20 (1.58–3.07) and 4.45 (3.47–8.54) in Japanese women, 1.61 (1.14–2.26) and 2.63 (1.79–3.88) in Egyptian women, 2.35 (1.88–2.95) and 8.45 (6.01–11.87) in Japanese men, and 2.33 (1.21–4.47) and 5.06 (2.52–10.17) in Egyptian men.

Older age was associated with poor self-reported health among Japanese men only after adjusting for family- and work-related factors. In reference to the age group ≤ 30 years, the multivariable OR (95% CI) for poor self-rated health was 1.40 (1.00–1.96) for the age group 41–50 years and 1.48 (1.03–2.13) for the age group > 50 years. However, no significant association between age and self-reported health was observed among Japanese women or Egyptian men and women. In general, we did not observe a significant association between smoking, alcohol drinking, or physical activity with self-rated health; however, Egyptian male current smokers tended to have poor self-rated health; the multivariable OR: 95%CI = 2.27:1.16–4.45 when compared to never smokers.

## Discussion

In this large cross-cultural study, we compared the prevalence of W_F_Cs and its two forms (FWC and WFC), factors associated with each form of conflict, and the association of these conflicts with the poor self-rated health status among Japanese and Egyptian civil workers. A higher prevalence of the conflicts was evident in Egyptians than in Japanese (except for a higher prevalence of FWC in Japanese than in Egyptian women), while a higher prevalence of poor self-rated health was estimated for Japanese than Egyptian civil workers of both genders. Work environment, family size, and being single was associated with the conflicts in both countries. On the other hand, cohabitants in Japan and education in Egypt were associated with conflicts between work and family. For both genders in both countries, the odds for rating one’s health status as poor was strongly related to the level of FWC, WFC, and total W_F_Cs. Even with longer working and commuting time for Japanese civil workers, their lower W_F_Cs score may imply a difference in family function's ideal or cultural expectation. On the other hand, the higher proportions of Egyptians than the Japanese counterparts selecting the highest conflict frequency option “often” may reflect actual differences in the conflict level or just represent cultural differences in choosing extreme choices.

### The prevalence and correlates of W_F_Cs

A study conducted among 1021 Egyptian community dwellers revealed that 46.7% had high WFC, and 50.8% had high FWC [12], based on a score greater than or equal to the median MIDUS score. *Burke and El-Kot *[19] found a higher mean W_F_Cs in 242 (146 males and 96 females) Egyptian managers than that in the US based on a different scale; i.e., Carlson et al. [30]. *Kobayashi *et al*.* reported the prevalence of high WFC and FWC among Japanese residents as 36.6% and 36.4% in men and 28.4% and 56.8% in women [8], based on the median MIDUS score. Based on an absolute cutoff point (11 points of MIDUS score), the prevalence of strong W_F_Cs estimated by *Koura *et al*.* was much higher; 54.0% in 1258 male and 72.5% in 550 female civil servants on the west coast of Japan [13]. In contrast, *Shimazu *et al*.* used a cutoff greater than or equal to the median of the Survey Work-home Interaction-NijmeGen (SWING) score [31] and reported that Japanese dual-earner couples with preschool children had lower prevalences of high WFC; 15.2% in males and 22.5% in females, and lower prevalences of high FWC; 21.2% in males and 16.3% in females [20]. The estimated prevalences in other working populations varied largely by culture, study design, and conflicts’ diagnostic tool. Still, they were generally comparable to our estimated prevalence and indicative of the higher prevalence in women than men. *Lallukka *et al*.* used the highest quintile value of the MIDUS score as a cutoff for strong versus weak categories of W_F_Cs and estimated the prevalence of high W_F_Cs as 17% in British male civil employees, 18% in Finnish male civil employees, and 19% in Japanese male civil employees, the respected prevalence among female civil employees of the three countries was 22%, 19%, and 20% respectively [5]. The prevalence of high W_F_Cs was 18% and 24% among full-time employed European men and women, according to the European Working Conditions Survey 2015 [24]. *Griep *et al*.* used a different scale divided into never, sometimes, and frequent conflicts and reported a prevalence of 16.0% for the frequent WFC and 7.5% for the frequent FWC among Brazilian men and 25.0% and 6.8% among Brazilian women [32].

*Byron* suggested that work-related factors are more associated with WFC than FWC, while some family factors are associated more with FWC than WFC [3]. The work-related factors in our study were associated, in both countries, with the WFC in both genders. This result was consistent with previous studies that working longer, overtime, and shift work [17–19, 21]*,* representing high job demands and low job control [33]*,* were positively associated with WFC. Workload could negatively affect workers who struggle to balance work demands and family roles.

On the other side, the family structure affected the Japanese civil workers’ perception of FWC. The results match previous findings in Japan [17, 18, 21] and Europe [34, 35]. Living with children adds significantly to FWC, and the effect is more substantial when the children are in the youngest age group. Similar to our results, *Fujimura *et al*.* reported an increased odd of high FWC among Japanese civil servants living with children; OR = 1.65, 95%CI: 1.33–2.05, and a decreased odd of FWC among those living with parents; OR = 0.86, 95%CI: 0.71–1.04 [21]. The null association between cohabitants and W_F_Cs in the Egyptian civil workers might be attributed to the high proportion of Egyptians living in multigeneration families in the same household; thus, the load of children in the family could be compensated by the presence of grandparents [36]. An interesting finding was that living with other family members was inversely associated with the Japanese women's but positively associated with the Egyptian women’s FWC. As polygyny is allowed in Egypt and was estimated at 4% among Egyptian men [37], it paved the way for jealousy and conflict when other family members in Egyptian women’s lives include co-wife, stepchildren, and co-sister-in-law [38], and might explain the positive association with the FWC. Unfortunately, the Egyptian survey did not capture the percentage of families having more than one wife.

Findings among Egyptian civil workers agreed with those from other working populations, where *Griep *et al*.* in Brazil [32] and *Carnicer *et al*.* in Spain [34] found higher education related to higher WFC. Highly educated subjects are expected to attain higher rank jobs with greater job demands, competitive edge, and expectations. The majority of civil workers in the Japanese cohort were educated to high levels (only 5.8% were junior high or high school graduates); this might explain why education did not show such association among the Japanese civil workers.

### The reported self-rated health status and its associations with W_F_Cs in both countries

Similar to our findings, the previous studies indicated a higher prevalence of poor self-rated health in Japanese; 13.9% in men and 17.7% in women [8], 35.2% in men and 36.0% in women [13] than Egyptians; 16.9% [12].

Despite the observed gender difference in the prevalence of W_F_Cs and poor self-rated health, our results are in line with the previous research showing associations of W_F_Cs and its two forms, WFC and FWC, with poor self-rated health of both genders in Japan [8, 13, 23], Egypt [12], and other populations [4, 22, 24, 32]. Increased inter-role conflicts between work and family domains can reduce the time for sleep [28] or leisure activities, thus increasing psychological stress [9] and affecting physical [3] and mental [11, 14, 15] health conditions.

### Strengths and limitations

The present study has several strengths and limitations. Due to the relatively large sample of recruited Egyptian and Japanese civil workers, there was no problem with the statistical power for the gender- and country-specific analyses in the current study. Also, the participants were working in a wide range of public occupational sectors rather than one-company samples. In addition, we did not assess the targeted variable (W_F_Cs) via a simple question; on the contrary, we used an expanded validated questionnaire that was widely used internationally and in both populations. Regarding the limitations, the cross-sectional design, convenience sampling, and self-reported data could have introduced some selection and misclassification bias. Thus, the causal inference and the generalizability of the results to the whole working population; nevertheless, those in the private enterprise are not possible.

## Conclusions

The prevalence and the factors related to W_F_Cs and self-rated health varied by gender among the Japanese and Egyptian civil workers. However, the robust associations between W_F_Cs and poor self-rated health were evident for male and female civil workers in both countries. Our findings suggest that governmental measures are required to improve the working environment of Japanese and Egyptian civil workers to prevent work-family role conflicts from increasing their risk of poor health. Interventions that focus on the family-related factors among Japanese civil workers are recommended. In contrast, more flexible work arrangements should be made available to the highly-educated Egyptian civil workers.

## Supplementary Information

**Additional file 1.**

## Data Availability

Data are available upon reasonable request to the corresponding author.
