# Supplementary material for: A comparative study of the work-family conflicts prevalence, their sociodemographic, family, and work attributes, and their relation to the self-reported health status in Japanese and Egyptian civil workers
Source: BMC Public Health. 2022 Aug 5;22:1490. doi: 10.1186/s12889-022-13924-0 (PMC9354292; doi:10.1186/s12889-022-13924-0)
Supplement: Supplementary file 1 — Additional file 1. [file 12889_2022_13924_MOESM1_ESM.pdf]

Supplemental Table 1. Gender- and country-specific civil workers' characteristics (mean ± standard deviation/proportion) [a] according to levels of work-family conflicts (W_F_Cs).

	Japan				Egypt			
	Levels of work-family conflicts (W_F_Cs)				Levels of work-family conflicts (W_F_Cs)			
	**Weak**	**Fair**	**Strong**	**P-trend[b]**	**Weak**	**Fair**	**Strong**	**P-trend[b]**
**Women**								
Number of subjects, n	859	447	291		752	479	383	
Total work-family conflicts (W_F_Cs) score	2.0 ± 1.5	6.0 ± 1.2	9.6 ± 1.8	<0.001	2.8 ± 2.1	8.1 ± 1.1	11.5 ± 1.7	<0.001
Family-to-work conflict (FWC) score	0.7 ± 1.1	3.0 ± 1.3	5.2 ± 1.5	<0.001	1.6 ± 1.4	3.6 ± 1.5	5.2 ± 1.4	<0.001
Work-to-family conflict (WFC) score	1.3 ± 1.2	3.0 ± 1.2	4.5 ± 1.4	<0.001	1.9 ± 1.5	3.8 ± 1.5	6.3 ± 1.4	<0.001
High education, %	67.3	56.4	63.2	0.22	65.6	68.9	70.0	0.41
Professional occupation, %	51.0	53.2	63.6	<0.001	63.3	68.5	69.5	0.23
Married, %	42.7	72.9	80.6	<0.001	77.4	78.9	77.8	0.33
Living alone %	13.5	10.5	4.5	<0.001	0.5	0.4	0.5	0.60
Number of family members	2.0 ± 1.4	2.4 ± 1.5	2.6 ± 1.3	<0.001	3.0 ± 1.8	3.2 ± 1.9	3.0 ± 1.9	0.98
Women with children <14 years, %	15.3	40.5	55.5	<0.001	47.5	54.7	55.9	0.01
Number of children <14 years	0.4 ± 0.8	0.9 ± 0.9	1.1 ± 1.0	<0.001	1.0 ± 1.3	1.1 ± 1.3	1.1 ± 1.2	0.08
Job hours per day	8.2 ± 1.2	8.1 ± 1.4	8.1 ± 1.6	0.05	6.6 ± 1.2	6.9 ± 1.6	7.2 ± 1.8	<0.001
Commuting time to work, min	47.3 ± 25.0	44.7 ± 23.3	43.4 ± 21.6	0.01	28.0 ± 20.9	30.9 ± 21.8	33.1 ± 22.5	0.001
Regular daytime work, %	75.9	76.0	73.1	0.01	92.2	81.0	70.8	<0.001
Working overtime/Extra job, %	9.9	13.7	19.6	<0.001	8.5	14.4	22.2	<0.001
Current smoker, %	2.1	2.0	2.1	0.07	0.4	0.4	0.0	0.10
Current alcohol drinker, %	68.3	64.9	64.6	0.52	NA	NA	NA	NA
Metabolic equivalent units	55.1 ± 10.1	54.3 ± 9.3	54.7 ± 9.0	0.15	47.5 ± 9.5	47.4 ± 10.1	47.7 ± 10.2	0.77
**Men**								
Number of subjects, n	2108	821	342		787	424	286	
Total work-family conflicts (W_F_Cs) score	1.8 ± 1.5	5.9 ± 1.2	9.3 ± 1.6	<0.001	2.6 ± 1.8	7.1 ± 1.1	11.5 ± 1.7	<0.001
Family-to-work conflict (FWC) score	0.6 ± 1.0	3.0 ± 1.2	4.9 ± 1.6	<0.001	1.3 ± 1.3	3.3 ± 1.5	5.2 ± 1.6	<0.001
Work-to-family conflict (WFC) score	1.2 ± 1.3	2.9 ± 1.1	4.4 ± 1.4	<0.001	1.9 ± 1.5	3.6 ± 1.5	6.4 ± 1.4	<0.001
High education, %	86.6	87.0	90.1	0.14	49.4	49.4	57.0	0.66
Professional occupation, %	43.6	42.0	48.0	0.39	48.0	46.7	49.0	0.65
Married, %	69.0	85.0	88.9	<0.001	92.0	90.3	86.0	0.75
Living alone%	9.9	3.4	3.5	<0.001	0.6	1.2	0.7	0.73
Number of family members	2.3 ± 1.4	2.6 ± 1.3	2.6 ± 1.3	<0.001	4.0 ± 1.8	3.9 ± 1.7	3.7 ± 1.9	0.05
Women with children <14 years, %	23.8	44.3	57.9	<0.001	57.2	65.1	61.9	0.02

Number of children <14 years	0.5 ± 0.8	0.9 ± 1.0	1.1 ± 1.0	<0.001	1.3 ± 1.4	1.5 ± 1.3	1.4 ± 1.4	0.04
Job hours per day	8.3 ± 1.0	8.5 ± 1.2	8.6 ± 1.5	<0.001	7.3 ± 1.6	7.7 ± 1.9	7.6 ± 1.9	<0.001
Commuting time to work, min	56.4 ± 26.5	55.6 ± 25.2	58.3 ± 26.4	0.50	30.5 ± 20.3	31.8 ± 22.6	31.9 ± 20.7	0.25
Regular daytime work, %	93.3	89.5	82.8	<0.001	81.7	75.7	70.3	<0.001
Working overtime/Extra job, %	10.4	17.9	30.4	<0.001	35.3	45.3	69.6	<0.001
Current smoker, %	12.6	11.9	12.6	0.97	10.7	4.3	12.6	0.19
Current alcohol drinker, %	83.1	83.9	79.2	0.26	NA	NA	NA	NA
Metabolic equivalent units	54.0 ± 8.8	54.5 ± 8.8	54.7 ± 8.9	0.15	48.4 ± 9.8	48.8 ± 9.8	50.1 ± 10.2	0.02

[a] Continuous variables were expressed as men ± SD and categorical variables as percentages.

[b] P-trend were calculated by age-adjusted linear regression for continuous variables and age-adjusted logistic regression for categorical variables.
